# The gut microbiota to the brain axis in the metabolic control

**DOI:** 10.1007/s11154-019-09511-1

**Published:** 2019-10-28

**Authors:** Estelle Grasset, Remy Burcelin

**Affiliations:** 1 Wallenberg Laboratory, Department of Molecular and Clinical Medicine, University of Gothenburg, 41345 Gothenburg, Sweden; 2 Institut National de la Santé et de la Recherche Médicale (INSERM), Toulouse, France; 3 Unité Mixte de Recherche (UMR) 1048, Institut des Maladies Métaboliques et Cardiovasculaires (I2MC), Team 2 : ‘Intestinal Risk Factors, Diabetes, Université Paul Sabatier (UPS), Dyslipidemia’, F-31432 Toulouse, Cedex 4 France

**Keywords:** Gut microbiota, Entero-endocrine hormones, Peripheral nervous system, Glucose, Diabetic neuropathy

## Abstract

The regulation of glycemia is under a tight neuronal detection of glucose levels performed by the gut-brain axis and an efficient efferent neuronal message sent to the peripheral organs, as the pancreas to induce insulin and inhibit glucagon secretions. The neuronal detection of glucose levels is performed by the autonomic nervous system including the enteric nervous system and the vagus nerve innervating the gastro-intestinal tractus, from the mouth to the anus. A dysregulation of this detection leads to the one of the most important current health issue around the world i.e. diabetes mellitus. Furthemore, the consequences of diabetes mellitus on neuronal homeostasis and activities participate to the aggravation of the disease establishing a viscious circle. Prokaryotic cells as bacteria, reside in our gut. The strong relationship between prokaryotic cells and our eukaryotic cells has been established long ago, and prokaryotic and eukaryotic cells in our body have evolved synbiotically. For the last decades, studies demonstrated the critical role of the gut microbiota on the metabolic control and how its shift can induce diseases such as diabetes. Despite an important increase of knowledge, few is known about 1) how the gut microbiota influences the neuronal detection of glucose and 2) how the diabetes mellitus-induced gut microbiota shift observed participates to the alterations of autonomic nervous system and the gut-brain axis activity.

## Introduction

The blood glucose level or glycemia is one of the most regulated physiological parameters. It has to be maintained around 5.5 mM whitin the day, including the fasting periods as well as the prandial and post-prandial periods. Insulin and glucagon are both pancreatic hormones playing a critical role in the regulation of glycemia and their secretion depend on an efficient glucose detection. On the first hand, variation of blood glucose level is directly detected by the pancreatic beta cell i.e. when the increased arterial glycemia favors glucose entry into the beta cell through the glucose transporter GLUT2, the phosphorylation of glucose by the glucokinase leads to the triggering of insulin exocytosis. On the other hand, the regulation of glycemia is under nervous influences 1) from the central nervous system, particularly the hypothalamus which detects glycemia variations throughout the day in the systemic blood and 2) from the autonomic nervous system including the enteric nervous system (ENS) and the vagus nerve (VN) which detect the glycemia variations within the gastro-intestinal tract, from the mouth to the colon, and the portal vein during post-prandial period. When a variation of glycemia is detected, all the nervous systems send neuronal efferent messages to the organs involved in glucose metabolism (peripheral organs) as the liver, the muscles, the pancreas and the adipose tissue to regulate the gluconeogenesis, the storage of glycogen or lipids, the secretion of hormones as insulin and glucagon resulting then to a stabilization of the glycemia.

Type 2 diabetes (T2D) is characterized by a dysregulation of glucose metabolism leading to fasting (>7 mM) and postprandial (>11 mM, 2 h after a meal) hyperglycemia. This dysregulation results from a large panel of different organ alterations notably an impaired insulin and glucagon secretion and action. Such impairments could originates from a lack of glucose awareness where hyperglycemia is not correctly detected by the body and therefore, glucose regulatory signals are not sent properly to the organs. During T2D, the neuron activities and homeostasis are highly impacted, particularly by the diabetic neuropathy, i.e. neurodegeneration induced by a long-time period of diabetic state, leading to a dysregulation of glucose detection and an inadequate response from the peripheral organs. It participates then to the disease aggravation.

For the last decade the role of gut microbiota in the regulation of glucose metabolism and the corresponding metabolic diseases as diabetes has been well-described. However, the molecular mechanisms involving the host/microbiota interactions require to be elucidated. Up to now, only few articles described the molecular impact of microbiota on the glucose neuronal detection. Therefore, we will 1) review how the glucose is detected by the nervous system; 2) discuss how the gut microbiota can influence it; 3) how we can use these microbiota-host interactions to improve the glucose detection during T2D and the efficiency of some therapies requiring the nervous system to act and to treat the diabetic neuropathy.

### Nervous glucose detection within the gut and its alteration during type 2 diabetes

#### Glucose-induced intestinal neuro-hormone secretions

**Fig. 1:**
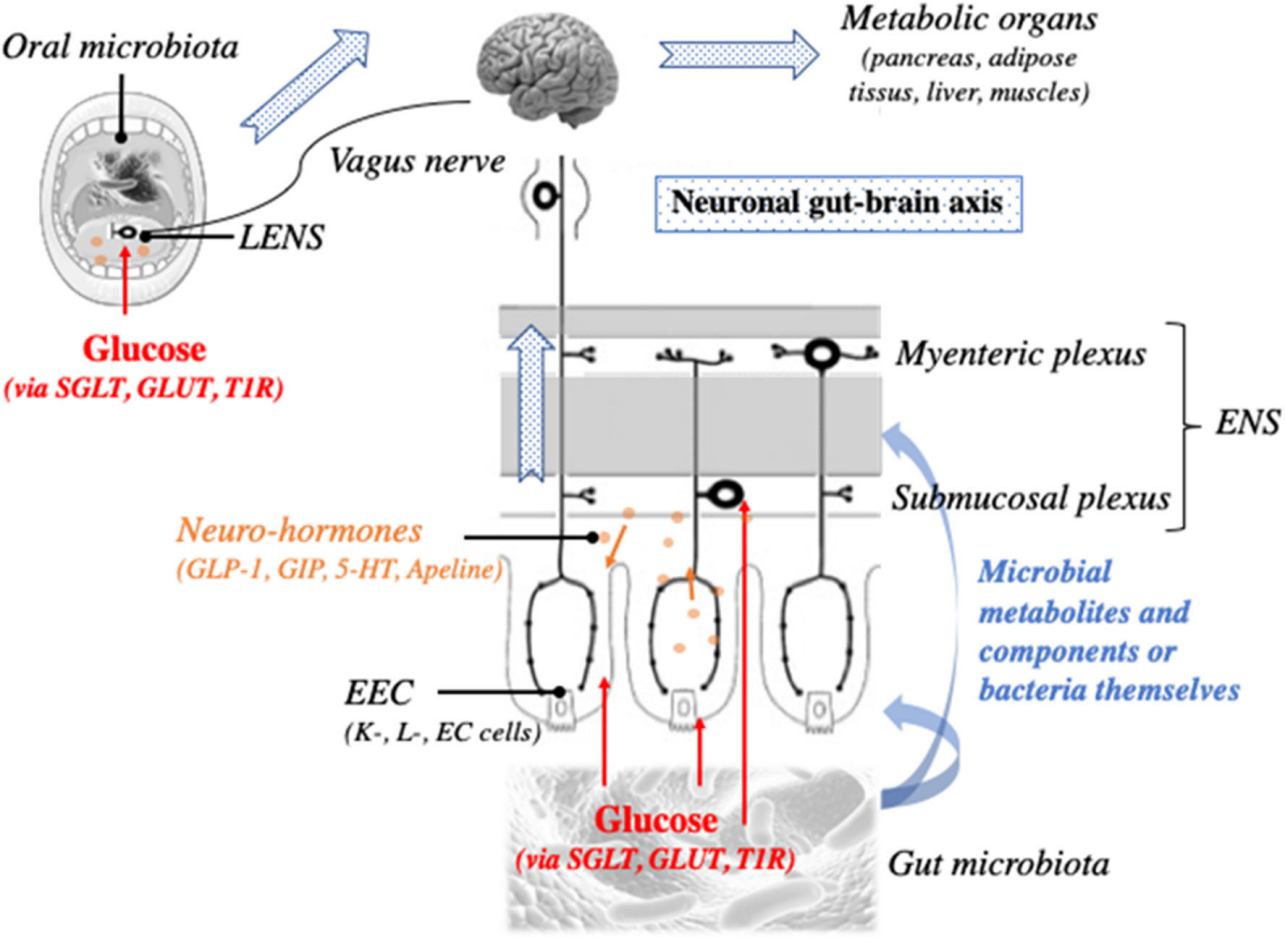
**The gut microbiota to the brain axis in the metabolic control**. The gut microbiota influences the intestinal system of glucose detection involving the entero-endocrine cells (EEC) and the intestinal neuro-hormones, the enteric nervous system (ENS) and the vagus nerve, through its metabolites, its components and some bacteria themselves. A neuronal glucose detection begins inside the mouth, in the gustative papilleae of the tongue innervated by the vagus nerve and the lingual enteric nervous system (LENS) and where reside also a prokaryotic community, the oral microbiota. The neuronal glucose detection is critical for the metabolism control

After a meal, entero-endocrine cells (EEC) within the intestinal mucosa secret a panel of intestinal hormones in response to nutritional and luminal glucose. It is detected in the luminal border of the EEC by specific receptors or transporters, namely the Taste Receptor 1 (T1R) and Sodium Glucose Cotransporter (SGLT) 1/3 [1–3] which induce secretion of intestinal neuro-hormones. Among them, there are Glucagon-Like Peptide 1 (GLP-1), Glucose dependent Insulinotropic Polypeptide (GIP), 5-hydroxytryptamine (5-HT) or serotonin respectively secreted by L and K cells and enterochromaffin cells [4, 5]. Apelin was also recently described as an intestinal hormone playing a critical role in metabolism regulation but the type of secreting cells is not identified yet [6, 7]. By immunohistochemistry, Wang et al. observed that the apelin immuno-signal colocalized with chromogranine A immuno-signal, which is a general marker of EEC, within the stomacal and ileal mucosa [7]. After their release, each neuro-hormone acts on a specific receptor express by the intestinal and portal innervations of the VN and the enteric neurons: GLP-1 on GLP-1 receptor, GIP on GIP receptor, 5-HT on 5-HT receptors 3 and 4 and apelin on APJ (Fig. 1).

During early stage of T2D, the intestinal genetic expressions of SGLT1 and GLUT2 are increased in rodents and humans as well as the secretions of intestinal neuro-hormones in response to glucose in prediabetic rat [8–10]. However, these compensatory mechanisms decrease with the diabetes duration in rodents suggesting a development of glucose resistance over time [11, 12]. During T2D, the neuro-hormone blood levels are particularly altered: the concentrations of GLP-1, GIP and 5-HT are maintained high revealing a resistance to the intestinal neuro-hormones as “incretin resistance” [12–15].

#### Vagus nerve, enteric nervous system and their alterations during type 2 diabetes

##### Within the intestine

The VN innervates the intestinal tractus and the portal vein. After a meal, it nervously detects the nutritional glucose reaching the intestinal tissue and the portal blood and informs the brain via a nervous axis called the gut-brain axis. Numerous studies showed that glucose infusions within the intestinal lumen and the portal vein controlled the food intake, the glucose consumption of liver and muscles and the endocrine secretion of pancreas through either sympathetic or parasympathetic (VN) innervations [16–19]. GLUT2 plays a critical role on the direct detection of glucose by the VN [16, 20]. However, the glucose detection can be indirect, i.e. through the intestinal neuro-hormones since the VN expresses also their specific receptors (Fig. 1). Thus, it detects directly the neuro-hormones after their release during the prandial and post-prandial periods ([21–24]. During a meal, the level of food intake plays a critical role on the neuro-hormone sensitivity since the nutrient levels influence the neuro-hormone receptor exposure in the vagal synapses [21, 24].

The ENS is an important network of neurons within the intestine. Its first described function was the regulation of the intestinal peristalsis involved in digestion processes [25]. Then, a lot of functions were attributed to the ENS as the control of the intestinal blood flow through the angiogenesis and the vessels motility; the intestinal immune system activities; the ion secretions within the lumen and the intestinal permeability [25]. The enteric neurons are organized in ganglions communicating with each other by innervations and divided in 2 plexi: the myenteric plexus localized between the inner and the outer muscular layers and involved in the control of intestinal transit and the submucosal plexus localized under the intestinal epithelium, within the lamina propria interacting with the blood vessels, the mucosa or the immune system [25]. Interesting studies demonstrated that glucose is detected through SGLT1 expressed by the enteric neurons, inducing then the phosphorylation of the protein Ca^2+/^calmoduline-dependant kinase II (CamKII) reflecting an activated neuronal state [26, 27]. Similarly, the intestinal neuro-hormones secreted in response to glucose, as GLP-1, 5-HT or apelin, activate enteric neurons and modulate the intestinal transit through their specific receptors [11, 14, 26–29]. Recent studies demonstrated that enteric neurons can play a critical role in the process of neuronal glucose detection (Fig. 1). When they are activated, they could transmit a neuronal message to the VN participating to the glycemia regulation through the gut-brain axis [14, 28, 30]. Furthermore, when the enteric neurons are pharmacologically destroyed, the GLP-1-induced gut-brain axis stimulation is decreased, impairing neuronal glucose detection [14]. Finally, during T2D, the vagal detection of the neuro-hormones is particularly altered [11, 14]. It was observed 1) fewer phosphorylation of CamKII, cell activation marker, in the EEC (enterochromaffin cells and L-cells), the enteric neurons and the nodose ganglia of the VN in response to glucose, in diabetic rats and 2) less neuronal expression of c-fos in response to glucose and GLP-1 in the brain stem of diabetic mice.

##### Within the tongue

The neuronal detection of glucose can be done before the small intestine, inside our mouth and particularly by the taste papillae, including the taste buds, within the tongue (Fig. 1). As it was observed in the EEC, the cells within the mucosa of taste papillae expresse the mRNA as well as the protein of T1R, GLUT1,3 and 4 and SGLT1 and expose them into the cell surface [31]. Endocrine cells secreting GLP-1 as L cells can be found inside the taste papillae and able to activate the VN [32]. The VN innervates the tongue but also some authors describe a sort of neuronal network comparable to the ENS within the gut called lingual ENS (LENS). The identified neurons are comparable to those involved in regulation of secretion and vasomotility within the gut. They can also regulate systemic reflexes on the digestive and respiratory apparatus through their innervations inside the pharynx or larynx. The LENS can modulate the activity of the annexed glands [33, 34]. Thus, the LENS and the VN innervating the tongue are the first neuronal network located at the beginning of the digestive apparatus which analyses the foods before their ingestion and diffuses this information distally (Fig. 1). It was recently demonstrated that the GLP-1 secretion by the taste buds is induced by the lipids trough CD36 and GPR120 and the aim is to regulate the palatability and the preference for the sweet water [35]. The stimulation of the taste buds with some long chain fatty acid induces a preference for the sweet water in a GLP-1r-dependant manner, since such behavior is not observed in the GLP-1r KO mice. Altogether few studies describes the role of the oral glucose detection in the regulation of glucose metabolism.

During T2D, the mechanisms involving the taste detection are modified. CD36 as well the gustudicine receptor are overexpressed in the taste buds of diabetic rats [36, 37] while their signaling, and particularly CD36 signaling, are altered [38] suggesting a mechanism of resistance for the nutrient detections within the tongue. An increase of apoptosis as well as a decrease of the innervation were observed by TUNNEL staining, western blot of BCL2, BAX and activated caspase 3 and 9 and immunostaining of PGP9.5, a neuronal marker in the taste buds of diabetic rats [39, 40]. Thus, these alterations of the tongue detection described by the decrease of the sensor cells or neuronal innervation inside the taste buds or an alteration of some intracellular signaling can induce an alteration of the food intake and food preferences leading to aggravate metabolic diseases.

##### Diabetic neuropathy

More than 50% of diabetic patients suffer from diabetic neuropathy. It is characterized by numerous alterations as axonal atrophies, demyelinisation, decrease of regeneration capacities, neuronal inflammation and an important decrease of distal, peripheral innervations called peripheral neuropathy. It could be considered as a nutritional neurodegenerative disease. These alterations participate to a worse neuronal conduction and a decrease of the amplitude of neuronal signals. A lot of mechanisms are involved as: 1) the intranerve angiopathy induced by a modification of vessel structure; 2) the production of glycated proteins, as glycated hemoglobin, increasing the blood viscosity and by chelation, limiting the nitric oxide biodisponibility affecting thus the vasomolitity; 3) a default of production and effects of neurotrophic factors induced by a intracellular stress of neurons and 4) the polyols flux modifying the function of glial cell as neuronal supporting cells, and affecting the electrical activity of neurons. Then, intranerve ischemia and oxidative stress induced by the polyol flux and glycated proteins are observed and participate the neurodegeneration process [41, 42]. A better knowledge about the involved mechanisms was permitted by studies on diabetic animal models as treated with streptozotocin, fed with high-fat diet, genetically-modified as *db/db* or *ob/ob* mice.

The diabetic neuropathy affects numerous organs giving rise to an intestinal transit and gastric emptying alterations (diarrhea and/or constipation; gastroparesis; nausea; …) [43], cardiovascular defaults (tachycardia, orthostatic hypotension, erectile troubles, etc), uncontrolled sweating, a hyper or hypo sensitivity during exercises or hormonal alterations. Despite of an important alteration of the quality of life, no efficient treatment is available in the pharmaceutical market. Associated to all these phenotypes, the diabetic neuropathy aggravates diabetes and alteration of glycemia regulation: gastroparesis and intestinal transit alteration affecting the nutrient entrance within the intestinal lumen and then into the blood, intestinal glucose malabsorption, a loss of nutrient detection by the neuronal gut-brain axis within the intestine as well as the portal vein, a default of energetic metabolism regulation in the central nervous system and in the periphery by a loss of neuronal conduction.

On the other hand, as diabetic neuropathy is a long-term complication of diabetes metillus, it occurs at the late stage of the disease and becomes even worst with aging [44]. By working on aging-associated neurodegenerative disorders, as Alzheimer or Parkinson, we can understand better as the neuronal homeostasis is critical for glycemia regulations. It is known that 30% of Parkinson patients have impaired glucose tolerance, among whom 5.6% were newly diagnosed T2D and 26% were insulin resistant [45, 46]. Inversely, the neurodegenerative disorders-suffering patients have more risk to develop obesity and T2D [47]. It was recently demonstrated that feeding a mouse models of Alzheimer disease (APP/PS1 transgenic mice) with an HFD induced a worsening of insulin and leptin resistance leading to an aggravation of the diabetic state [48]. A possible cause could be related to the mechanisms of the neurodegenerative process on the autonomic nervous system altering then the glucose detection. An atrophy of the VN was observed during Parkinson diseases [49] while a delay of vagus somatosensory evoked potentials or a dysfunctional nucleus tractus solitaries (the nucleus of the VN localized in the brain stem) are observed in Alzheimer’s disease [50, 51]. On the other hand, the ENS is also impacted. In APP/PS1 transgenic mice, the number of neuronal nitric oxide synthase (nNOS)-positive and acetylcholine transferase-positive neurons are decreased within the intestine [52]. During Parkinson disease, it is observed gastroparesis and intestinal transit alterations [53–55]. It could be due to an abnormal accumulation of α-synuclein inside the ENS and functional and neurochemical changes of the gastrointestinal tract [54–57]. Finally, taste sensations are highly impacted by all neurodegenerative disorders [58, 59].

#### Intestinal neuro-hormone and their potential role on neurogenesis/neuroprotection

Several studies observed that some intestinal neuro-hormones, whome are secreted in response to glucose, have neuroprotection and neurogenesis potentials as GLP-1 or 5-HT. GLP-1 induced axonal formation in primary culture of neurons from dorsal root ganglion (sympathetic branches of the autonomic nervous system) [60]. In a diabetic context, in streptozotocine-treated rats, a chronic treatment with GLP-1 or a GLP-1r agonist, exendin 4, improved the innervation within the periphery and the motor nerve activity (sciatic nerve) through a phosphoERK1/2 signaling [61]. A similar effect was observed in another animal model of sciatic nerve impairment [62]. By using serotonin transporter (SERT) inhibitor treatments, some authors observed that 5-HT had a similar effect on diabetic neuropathy [63, 64]. Within the intestine, liraglutide, a GLP-1 receptor agonist blocked lipopolysaccharide-induced visceral allodynia, which may be a -nitric oxide dependent response [65]. GLP-1, as well as GLP-2, significantly and concentration-dependently enhanced neuronal survival of myenteric neurons [66]. In other hand, 5-HT, through the 5-HT4r, is also able to promote the neuronal survival and maturation of myenteric neurons [67].

The neuroprotective effect can be indirect, through an effect on microglia and astrocytes. They expressed GLP-1r or GIPr and both hormones, GLP-1 and GIP, improved their survival, induced the neuronal growth factors expression, GDNF and NGF and reduced the oxidative stress [68].

In different animal model of neurodegenerative diseases, similar results were also observed. For example, in an animal model of neurodegenerative disease induced by ATP depleting-drug glucosamine, affecting the neuron metabolism and signaling, exendin 4 protected against the drug effect through an EPAC mechanism [69]. On the other hand, liraglutide, lixisenatide, as GLP-1r agonists able to cross the blood-brain barrier and a GIPr agonist reduced amyloid plaques, neurofibrillary tangles, the induced inflammation as well as stimulated neuronal progenitor proliferation and neurogenesis in a mouse model of Alzheimer [70–72]. Similar effect was observed in a mouse model of Parkinson disease [73]. 5-HT is also able to improve neurodegenerative state during Alzheimer disease through the 5-HT4r ([74].

Through their neuroprotective actions, the neuro-hormone involved in glucose detection seems to be important to send a neuronal message indicating that glucose is coming in prandial period but also to protect the nerve and neurons involved in this neuronal sending.

### Microbiota-host interactions involved in the nervous control of glucose metabolism

#### Microbiota and metabolism

From the mouth to the large intestine, a large, rich, diverse and complex prokaryotic community live in straight contact with the host (Fig. 1). Between 2000 and 2010, numerous studies pointed out the importance of the microbiota in the energetic metabolism regulation. It was showed that germ-free (GF) mice are leaner and have a better glucose tolerance than their conventionally-raised counterpart (Conv-R). The colonization of GF mice with an intestinal microbiota coming from a Conv-R resulted in a worse insulin tolerance and a 60%-increase of body fat induced by a higher lipogenic activity within the liver [75–77].

The microbiota can interact with the host through different mechanism (Fig. 1). By digesting fibers and nutrients, the microbiota can produce different molecules as 1) short chain fatty acids (SCFA) with the most well-known byturate, propionate and lactate but also succinate 2) indole and its derivatives, 3) some neuro-hormones or mimetics as GABA [78], ClpB (αMSH mimetic) [79], and 4) others molecules affecting the host as imidazole propionate [80]. The microbiota also modifies and transforms the biliary acids secreted in the intestinal lumen to secondary biliary acids by deconjugation, dehydrogenation, dihydroxylation or epimerization [81]. One the other hand, the microbiota interacts through its own components as lipopolysaccharides (LPS) or RNA and DNA fragments through the transmembrane and cytosolic receptors TLR and as peptidoglycanes through the receptor NOD. During the last decades, numerous studies contributed to improve the knowledge about the microbiota-host interactions particularly in the metabolism context. It was shown 1) that LPS, through TLR4 and CD14, participates to the low-grade inflammation observed during the metabolic diseases and aggravating the diseases and it was called endotoxemia [82, 83] and peptidoglycans through NOD2 can modulate colonization and intestinal inflammation influencing the sensitivity to insulin [84]; 2) the importance of dietary fibers and the SCFA production in the metabolism regulation through a GLP-1-dependant mechanism [85–88] and 3) the role of secondary biliary acid through TGR5 and FXR [81, 89, 90]. More recently, it was observed that succinate is a critical molecule produced essentially by Prevotella in the regulation of glucose metabolism and the weight in mice [91]. Also, it was observed that *E. coli* produces ClpB, an αMSH mimetic, controlling then the food intake [79]. Indole and its interaction with the receptor AhR plays also a critical role in the regulation of intestinal immune system activity and glucose metabolism through a GLP-1-dependant mechanism [92]. Similarly, a study observed that Akkermansia, a mucin-degradating bacteria, and particularly a membrane protein, prevented obesity and associated complications in mice [93]. Finally, recently, a new bacterially-produced molecule in a context of type 2 diabetes, imidazole propionate, was identified to modulate the liver activity and impair glucose metabolism [80].

#### Relation between the microbiota and the entero-endrocrine and nervous systems

The EEC, VN and ENS express different receptors or channels able to recognize either bacterial components or molecules produced by the microbiota (Fig. 1). The receptors TLR binding to LPS and some bacterial DNA and RNA fragments [94, 95], the FFAR binding to SCFA [96] or TGR5 binding secondary biliary acids LCA and DCA [97] to cite a few are expressed by these tissues. Interestingly, inside the taste buds of the tongue, TLR and TGR5 are expressed also [98, 99].

It is known that microbiota influence the neuronal activities modifying then the transit, regulating the neuronal homeostasis particularly the critical ratio neuron/glial cell [100–102]. To go further, GF mice as well as antibiotic-treated mice have an immature ENS with a weak basal activity. Their ENS, as their VN, cannot be activate by some neuro-hormones like GLP-1 [14, 103–105]. The colonization of GF mice with a normal and healthy gut microbiota restores homeostasis and neuronal activities of ENS and VN ([14, 103–105]. It is not true if the gut microbiota comes from diabetic mice [14].

Some lactobacilli (*L. reuteri* and *rhamnosus*) improve ENS and VN activations [106, 107] while others (*L farciminis, plantarum and fermentum*) can produce a neurotransmitter, the nitric oxide, and thus control the intestinal transit [108, 109]. The bacterially-produced nitric oxide can also influence the neuronal response to GLP-1 and thus the glucose metabolism [14]. It is recently showed that Bacteroides can produce another intestinal inhibitory neurotransmitter, GABA [78]. The microbial influence on the ENS homeostasis and activity can be mediated by TLR4 [100]. NOD2 as TLR4 are critical for the ENS sensitivity to the intestinal neuro-hormone as GLP-1 [14]. In the tongue level, LPS, through TLR4, decrease the neuronal response of taste buds to saccharose [99].

Another kind of microbial influence should be more investigated. It is recently observed that SCFA can modulate epigenetics of the cells of the liver, adipose tissue and the colon [110]. On the other hand, epigenetics is critical for the neuronal homeostasis and activity. For example, MeCP2 (methyl CpG bonding protein) is a protein influence by epigenetic modifications and a mediator of synaptic development and plasticity. An alteration of MeCP2 expression induces Rett syndrome in humans characterizing by important neuronal deficiency but also because it is expressed by enteric neurons [111], an important dysregulation of the intestinal transit and nitric oxide production [112]. Since nitric oxide is an important mediator of GLP-1 action (14) for the control of glucose metabolism, MeCP2 could influence glucose metabolism. On the other DNA methylation is also critical for the expression of N-myc Downstream-Regulated Gene 4 (NDRG4). This protein is critical for brain morphogenesis through BDNF production and neurites outgrowth and myelinisation. Its expression level is reduced in Parkinson disease and this protein is expressed in the enteric neurons, particularly the nNOS-positive neurons [113]. The other epigenetic modification is histone deacetylations. Histone deacetylase (Hdac) can be critical for neuronal homeostasis and plasticity [114]. Hdac6 modulate alpha-tubulin, a critical protein in axon formation, expression [115]. Hdac dysregulation are also observed in neurodegenerative disorders [116–118]. Interestingly, it was observed that Hdac6 inhibition protects against vincristine-induced peripheral neuropathy [115]. Thus, by understanding better the microbiota-induced epigenetic modifications within the neurons of the VN or of the intestine could increase the knowledge about the molecular link between microbiota and neurons.

Finally, a recent work demonstrated that the influence of gut microbiota on the ENS and the VN homeostasis and activity can then influence the regulation of glucose metabolism [14].

#### The microbiota during type 2 diabetes and other neurodegenerative diseases

In humans as well as in animals, an important dysbiosis occurs during metabolic diseases, obesity and diabetes first described as an increase of the ratio Firmicutes/Bacteroidetes [119–122]. The gut microbiota impoverishes of Gram+ bacteria or bacteria producing SCFA as butyrate and gets rich of pathogenic bacteria [83, 123, 124]. Numerous authors demonstrated that the dysbiose can be a critical mechanism involved in metabolic disease development since the colonization of GF mice with a gut microbiota coming from diabetic/obese animals induce metabolic disease, despite of a normal chow diet [119, 125]. On the other hand, the bacteria involved in a better glucose metabolism, as Akkermensia, are reduced during obesity or diabetes [126]. Thus, target the microbiota with specific treatment as prebiotic or probiotic is an option widely studied around the world to treat metabolic diseases.

An important mouth dysbiosis is also observed. This dysbiosis induces dental pathologies observed during obesity and T2D but also influence the plasma cholesterol level, a blood parameter altered during metabolic diseases [127–130]. Some authors recently showed that *Porphyromonas gingivalis*-induced periodontitis is able to modulate immune system and promote glucose intolerance in mice fed with a high fat diet [131]. However, a lot is currently missing about the exact role of mouth dysbiosis in the metabolic alterations observed during type 2 diabetes.

The influence of the intestinal dysbiosis on the neuronal glucose detection was recently showed. The dysbiotic microbiota alters the neuronal homeostasis and the neuronal activities of the gut-brain axis in response to GLP-1 for the glucose metabolism regulation. These alterations can be transferred to GF mice when they are colonized with the flore coming from diabetic mice. Interestingly, an antibiotic treatment abolishes these alterations [14]. To go further, a probiotic treatment using different Lactobacilli is enough to restore the GLP-1 sensitivity in diabetic mice (data not published).

These set of data demonstrate clearly the importance of a good neuronal communication and it is highly influence by the gut microbiota and its metabolism. In neurodegenerative disorders, data showed that patients have an important dysbiosis [53, 132, 133] and it can influence the state of diseases. It was shown that 1) the presence of microbiota is determinant for the over-expression of alpha-synuclein; 2) colonization of GF mice with a gut microbiota coming from a Parkinson patient is able to induce motor dysfunction in mice and 3) SCFA plays a critical role in the pathology [134]. Along the same way, it was observed that a mouse model of Alzheimer disease, the APP/PS1 transgenic mice, without gut microbiota showed less beta-amyloid plaque formation and its colonization with gut microbiota coming from APP/PS1 transgenic mice is worst in term of beta amyloid plaque formation compared to its colonization with a gut microbiota coming from healthy mice [135].

### Current treatment of type 2 diabetes: How can we improve their efficiency

Some anti-diabetic treatments require a high efficiency of the autonomic nervous system as for example, the GLP-1 based therapies as dipeptidyl peptidase 4 (DPP-4, “gliptine”) inhibitors or GLP-1 analogues (“tides”). GLP-1 acts in different organs involved in the regulation of glucose metabolism: the pancreas by stimulating the insulin secretion and by inhibiting the glucagon secretions; the stomach and the intestine by inhibiting the gastric emptying; intestinal absorption, and the intestinal transit; and the brain by decreasing the food intake. The most described and important pathway involved requires the autonomic nervous system and the neuronal gut-brain axis. It recruits the vagal innervations within the intestine and portal vein walls. Indeed, the nodose ganglions of the VN expressed the GLP-1r [22, 23]. When the GLP-1r gene is specifically deleted in the nodose ganglion, it induces a dysregulation of glucose metabolism, particularly the food intake, the gastric emptying and the insulin secretion [136]. Animal experiences testing the effect of mechanically-induced or chemically-induced vagotomy [14, 137, 138] as well as measuring electrophysiologically the VN activity induced by GLP-1 agonist or DPP-4 inhibitors [137, 139] demonstrated clearly the importance of the neuronal message sent to the brain by the VN. Additionally, a recent study suggests an important intermediate role of the ENS in this neuronal message since the chemically induced-ENS neuropathy alters the GLP-1 actions as observed in vagotomized animals [14]. However, the role of the ENS in the regulation of glucose metabolism should me more studied and explored. It could be interesting to study the impact of enteric neuropathy in the regulation of glycemia. Different animal models are available to answer this question as: 1) chemically-induced enteric neuropathy [14], 2) genetically-induced enteric neuropathy [140, 141], 3) gliopathy-induced enteric neuropathy [142]. In troncally-vagotomized-humans, the glucose metabolism is highly altered and characterized by dysregulations of gastric emptying and glucose-induced insulin secretion [143]. All the later data suggest the important role of the neuronal gut brain axis in the efficiency of anti-diabetic treatments. Furthermore, a recent study demonstrated that a co-treatment with neurturin, a glial cell-line derived neurotrophic factor, with a GLP-1 analogue improved the diabetic state of Zucker fatty rats [144].

The bariatric surgery, whose effectiveness on metabolic diseases is well-accepted and described, have a positive effect on the glucose metabolism through an important GLP-1 effect but also an important modification of the gut microbiota [145]. The VN could be involved in the beneficial effects observed after the surgery [146]. However, the real effect of bariatric surgery on the autonomic nervous system, including the VN or the ENS, and the diabetic neuropathy is not described yet and requires further experiments. According to the type of undergone surgery and the tissue analyzed post-surgery, the vagal innervations can be differently impacted: it was observed an important apoptosis of the vagal neurons for some surgeries while for the others, it was observed an important neurogenesis of these neurons [147]. No publications studied the long-term impact of the bariatric surgery on vagal or enteric neuron activities, homeostasis, neurogenesis or apoptosis processes or the innervation level of the gut and other tissues. It could be interesting to study further the impact of these surgery on the neuronal state and understand the role played by the gut microbiota.

The fecal microbiota transplantation (FMT) showed also an important and efficient effect to treat some diseases. From the beginning, this therapy was used to treat *Clostridium difficile* infection inducing hemorrhagic diarrhea and an important bowel inflammation. However, some research group try to extend this therapy in other diseases as metabolic diseases. It was also recently demonstrated that lean donor fecal microbiota transplantation in obese metabolic syndrome patients was efficient to treat insulin resistance through bacterial metabolites modification. However, the efficiency of the treatment is dependent of baseline fecal microbiota composition [148]. On the other hand, in a mouse model of Parkinson diseases, the FMT showed a neuroprotective effect through a suppression of the TLR4/TNFα pathway [149]. A beneficial effect of FMT through a better the gut-brain activity is not explored yet and we should go further to understand the involved mechanism.

The diabetic neuropathy is an important feature that needs to be diagnosed before applying a treatment to a diabetic patient since it can influence the efficacy of the treatment. Thus, research on alternative or co-treatment is required and necessary. As the microbiota dysbiosis can alters neuronal activities and homeostasis, by treating this dysbiosis could improve first the neuropathy state by also improve the efficiency of some current anti-diabetic treatment.

## Conclusion

This review highlights the importance of autonomic nervous system, including the VN and the ENS, 1) on the physiological mechanism involved in glucose detection and glycemia regulation, from the mouth to the colon; 2) during the metabolic diseases either in the aggravation mechanisms or in the efficiency of some anti-diabetic treatment. Nevertheless, no efficient treatment is currently available to treat diabetic neuropathy. Diabetic neuropathy occurs after a middle to a long-time period of diabetes metillus (type 1 and type 2) and is usually associated to the aging. Along the same line, aging is also characterized by an increased risk of neurodegenerative diseases. Few is known about the problem of glucose metabolism during neurodegenerative diseases.

This review also proclaims how the gut microbiota is critical either in the neurogenesis, neuroplasticity and neuronal activities or neuronal alteration observed during T2D or neurodegenerative diseases. New studies are required to better understand the molecular mechanisms involved in the relation between microbiota and neuronal tissues. Thus, pro and pre-biotic treatment could be used to treat neuropathy or improve the current anti-diabetic treatments. Recent clinical trials demonstrated the role of dextrin [150], inulin-enriched oligofructose [151] or inuline alone [152] as well as L. acidophilus, Bifidobacterium animals [153, 154] and showed that these molecules improved glycemia control, insulin sensitivity and low-grade inflammation. However, none of these studies relate the effect on diabetic neuropathy. On the other hand, a lot of new animal works showed that current anti-diabetic treatment as metformin, GLP-1 agonist, DPP-4 inhibitors can positively modify the gut microbiota improving then the glucose metabolism [155–157]. However, although these anti-diabetic therapies induced an improvement of diverse neuropathies, no results permit to demonstrate how the gut microbiota shifts induced by the same therapies could influence and improve the neurons homeostasis and activities. Thus, this review suggests new mechanisms by how the gut microbiota influences glucose metabolism and opens new perspectives on how we can better treat T2D .
